# Cannabinoid Analgesia in Postoperative Pain Management: From Molecular Mechanisms to Clinical Reality

**DOI:** 10.3390/ijms25116268

**Published:** 2024-06-06

**Authors:** Antonio J. Carrascosa, Francisco Navarrete, Raquel Saldaña, María S. García-Gutiérrez, Belinda Montalbán, Daniela Navarro, Fernando M. Gómez-Guijarro, Ani Gasparyan, Elena Murcia-Sánchez, Abraham B. Torregrosa, Paloma Pérez-Doblado, Luisa Gutiérrez, Jorge Manzanares

**Affiliations:** 1 Servicio de Anestesiologia y Reanimación, Hospital Universitario 12 de Octubre, Avda. Córdoba s/n, 28041 Madrid, Spain; acarras3@hotmail.com (A.J.C.); rasalca@gmail.com (R.S.); belinda.montalban@gmail.com (B.M.); fernando_guijarro@yahoo.es (F.M.G.-G.); emurcias@gmail.com (E.M.-S.); pdoblado1@hotmail.com (P.P.-D.); 2 Instituto de Neurociencias, Universidad Miguel Hernández-CSIC, Avda de Ramón y Cajal s/n, San Juan de Alicante, 03550 Alicante, Spain; fnavarrete@umh.es (F.N.); maria.ggutierrez@umh.es (M.S.G.-G.); dnavarro@umh.es (D.N.); agasparyan@umh.es (A.G.); a.bailen@umh.es (A.B.T.); luisa.gutierreze@umh.es (L.G.); 3 Redes de Investigación Cooperativa Orientada a Resultados en Salud (RICORS), Red de Investigación en Atención Primaria de Adicciones (RIAPAd), Instituto de Salud Carlos III, MICINN and FEDER, 28029 Madrid, Spain; 4 Instituto de Investigación Sanitaria y Biomédica de Alicante (ISABIAL), 03010 Alicante, Spain

**Keywords:** cannabinoid, analgesia, postoperative pain

## Abstract

Postoperative pain (POP) is a challenging clinical phenomenon that affects the majority of surgical patients and demands effective management to mitigate adverse outcomes such as persistent pain. The primary goal of POP management is to alleviate suffering and facilitate a seamless return to normal function for the patient. Despite compelling evidence of its drawbacks, opioid analgesia remains the basis of POP treatment. Novel therapeutic approaches rely on multimodal analgesia, integrating different pharmacological strategies to optimize efficacy while minimizing adverse effects. The recognition of the imperative role of the endocannabinoid system in pain regulation has prompted the investigation of cannabinoid compounds as a new therapeutic avenue. Cannabinoids may serve as adjuvants, enhancing the analgesic effects of other drugs and potentially replacing or at least reducing the dependence on other long-term analgesics in pain management. This narrative review succinctly summarizes pertinent information on the molecular mechanisms, clinical therapeutic benefits, and considerations associated with the plausible use of various cannabinoid compounds in treating POP. According to the available evidence, cannabinoid compounds modulate specific molecular mechanisms intimately involved in POP. However, only two of the eleven clinical trials that evaluated the efficacy of different cannabinoid interventions showed positive results.

## 1. Introduction

Pain is an unpleasant sensory and emotional experience associated with, or similar to, actual or potential tissue damage [1]. It can be classified into two main categories based on the neurophysiological mechanisms underlying its origin: nociceptive pain, which arises from the activation of nociceptors in response to tissue damage caused by physical or chemical agents like trauma, chemical burns, or surgical procedures [2], and neuropathic pain, which results from direct damage or dysfunction of the sensory nerves [3]. Postoperative pain (POP) is a unique entity that necessitates prompt and effective alleviation to minimize suffering, facilitate the healing process and rehabilitation, and prevent complications. It is not solely attributable to inflammation or isolated nerve injury; its pathophysiology is unique, with specific consequences [4,5]. As a result, the response to analgesic treatment differs from other pain models. 

To date, opioids remain a mainstay of perioperative pain management, employed for sedation during general anesthesia, supplementation during regional anesthesia, and the treatment of acute postoperative pain [6,7,8]. However, despite their numerous benefits, opioids are associated with a well-documented adverse effect profile that can impede recovery and the resumption of daily activities. Moreover, the escalating rates of opioid consumption and misuse in the United States have reached epidemic proportions over the past decade [9,10]. Additionally, balancing adequate postoperative pain relief while mitigating the risk of overdose or relapse is challenging, particularly as a growing number of surgical patients exhibit opioid tolerance, such as those with chronic pain conditions [11]. Therefore, the effectiveness of opioids for pain management during the perioperative period is undergoing reevaluation [12]. 

The search for drugs that offer advantages over existing prescriptions is a fundamental goal of pharmacological research in managing POP. Thus, it is interesting to study families of analgesics with new mechanisms of action, high potency, and minimal undesirable effects. The incorporation of non-opioid adjuvant medications into a perioperative pain management plan can not only potentially enhance patient outcomes but also serve as a critical component in minimizing opioid utilization and potentially mitigating the downstream risk of opioid misuse and dependence.

In this context, there has been a renewed interest in cannabinoids, a class of compounds with a historical precedent for pain management dating back to the 19th century [13]. This resurgence can be attributed to the discovery of cannabinoid receptors [8,14] and endogenous substances that modulate these receptors [15,16], despite the inherent challenges in studying substances prone to abuse which are thus subject to stringent legislative control [17]. Arguments supporting their use include the following: (1) there is evidence of activation of the ECS after surgery [18,19]; (2) cannabinoids are analgesics per se; and (3) they are substances that can act as adjuvants to facilitate the analgesic action of other drugs. Nevertheless, despite experimental evidence demonstrating their antinociceptive efficacy, clinical outcomes have been constrained by the short duration of studies, small sample sizes, absence of control groups, and biases prevalent in most conducted studies. 

This review examines the current scientific evidence regarding the use of cannabis for the treatment of acute POP. Its interest lies in the fact that the etiology and treatment of pain produced by surgery are different from those of other painful conditions. Effective and rapid pain management is crucial to minimizing patient suffering and mitigating the complex physiological stress response triggered by surgery.

## 2. Cannabinoids and Therapeutic Potential in Pain Relief

**Figure 1:**
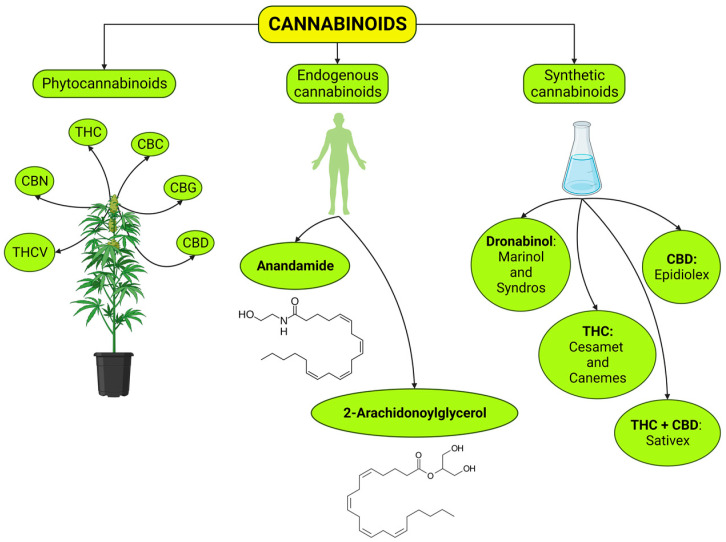
Classification of cannabinoid compounds according to their origin. THCV: Δ9-tetrahydrocannabivarin; CBN: cannabinol; THC: Δ9-tetrahydrocannabinol; CBC: cannabichromene; CBG: cannabigerol; CBD: cannabidiol.

Cannabinoids are a group of chemical substances derived from the cannabis plant, the endogenous cannabinoid system, or synthetic production that bind to varying degrees to cannabinoid receptors, including cannabinoid receptor type 1 (CB1r) [15,20,21,22,23] and type 2 (CB2r) [15,24,25,26,27,28,29,30,31,32]. This group of substances is extensive and diverse and can be classified in several ways. According to their origin, we distinguish three types of cannabinoids (Figure 1): (1) phytocannabinoids (naturally derived from plants); (2) endocannabinoids (endogenous cannabinoids); and (3) synthetic cannabinoids (artificially produced phytocannabinoids).

### 2.1. Phytocannabinoids

Phytocannabinoids are compounds characterized by a carbocyclic structure whose central ring is usually tetrahydropyran and by two chiral centers [33]. These compounds are produced naturally in the trichomes of the hemp plant (*Cannabis sativa*) to protect it against pests and the effects of the environment [34,35]. This plant is a tall annual shrub that grows naturally in temperate and tropical regions. It has been extensively utilized for medicinal purposes for millennia in various parts of Asia, particularly in India and China [36,37,38]. The resin extracted from the plant is known as hashish, while the name “marijuana” is attributed to the preparation of dried leaves and flowers of the plant.

More than 100 phytocannabinoids have been described, including their acid and neutral forms, analogs, and other transformation products [33,39]. The primary cannabinoids are ∆9-tetrahydrocannabinol (delta-9-THC or THC), 8-tetrahydrocannabinol (8-THC), cannabidiol (CBD), and cannabinol (CBN). Additionally, the plant contains other cannabinoids such as cannabichromene (CBC), cannabicyclol (CBL), cannabigerol (CBG), cannabigerol monomethyl ether (CBGM), cannabielsoin (CBE), cannabinodiol (CBND), cannabitriol (CBT), dehydrocannabifuran, and cannabicitran. The presence and quantities of these cannabinoids vary depending on the specific variety of *Cannabis sativa* being assessed [40]. It is interesting to note that *Cannabis sativa* synthesizes phytocannabinoids exclusively in their non-psychoactive acidic forms. Notably, the carboxyl group attached to these precursors is unstable and readily decarboxylates, releasing CO_2_ under heat or light exposure. This decarboxylation process transforms acidic cannabinoids into active neutral forms [41,42]. Furthermore, the relative abundance of each phytocannabinoid within the plant is significantly influenced by several factors, including growing conditions and extraction methods [19]. Additionally, cannabis has a complex botanical composition that encompasses over 200 terpenes and terpenoids. These terpenoids possess diverse pharmacological properties and have been linked to various therapeutic effects [43]. 

#### Phytocannabinoids and Pain Relief

THC remains the most extensively researched phytocannabinoid due to its potent psychoactive properties [44] and well-documented antinociceptive effects [45]. However, at higher doses, THC can induce intoxication. Despite this limitation, clinical trials generally support the efficacy of THC in managing chronic pain [46,47,48]. 

There are conflicting opinions in the literature regarding the use of phytocannabinoids for pain relief: (1) cannabis has been employed for medicinal purposes for millennia and the combination of its phytocannabinoids is more effective than currently available cannabinoid drugs. In this regard, emerging evidence suggests that herbal cannabis exhibits analgesic effects in both nociceptive and neuropathic pain. Notably, at least five high-quality randomized controlled clinical trials (RCTs) have demonstrated the efficacy of smoked cannabis in achieving pain relief [49,50,51,52,53,54]. (2) There is accumulating evidence and ongoing research for addressing common symptoms and conditions linked to pain, such as spasticity associated with multiple sclerosis or stroke [55,56], anxiety and posttraumatic stress disorder [57], migraine [58], nausea and vomiting [59], cachexia, inflammatory bowel diseases [60], and sleep disturbance [61,62]. (3) Cannabis has little capacity to cause overdose and is associated with lower rates of addiction compared to opioid analgesics. (4) Cannabis cultivation and production are relatively cost-effective. In contrast, there are detractors to the use of phytocannabinoids based on the following: (a) Herbal cannabis presents a complex chemical composition with significant variability and incomplete characterization, posing challenges for standardized dosing and consistent prediction of effects. Notably, cannabinoids can exhibit a spectrum of actions, sometimes even opposing effects, depending on several factors. These factors include the specific compound under investigation, its enantiomeric form, the plant species utilized in the study, and the patient’s overall health status [63,64,65]. As a result, it fails to meet the FDA criteria for drug approval. (b) Widespread recreational cannabis use raises concerns for potential individual and public health risks [66,67].

Consequently, there is an inherent risk that the availability of cannabis as a medicinal product will lead to increased accessibility and associated damage. (c) Only a small number of patients can achieve satisfactory clinical management; the advocacy for medical cannabis forms part of a well-structured and funded strategy to legalize cannabis for general use. (d) Inhaled cannabis via combustion methods, such as smoking, may present health risks due to the generation of harmful by-products. Thus, although inhaled cannabinoids produce bronchodilation, it is essential to note that the combustion of cannabis generates harmful by-products, including carbon monoxide, bronchial irritants, and potential carcinogens—the tar in a cannabis cigarette contains even higher concentrations of benzanthracenes and benzopyrenes [68]. Furthermore, some studies indicate that smoking cannabis, due to its consumption through deep and prolonged inhalations without a filter and higher combustion temperature than tobacco, may result in a fivefold elevation in carboxyhemoglobin levels and a threefold increase in tar intake compared to tobacco cigarettes [69].

### 2.2. Endocannabinoids

**Figure 2:**
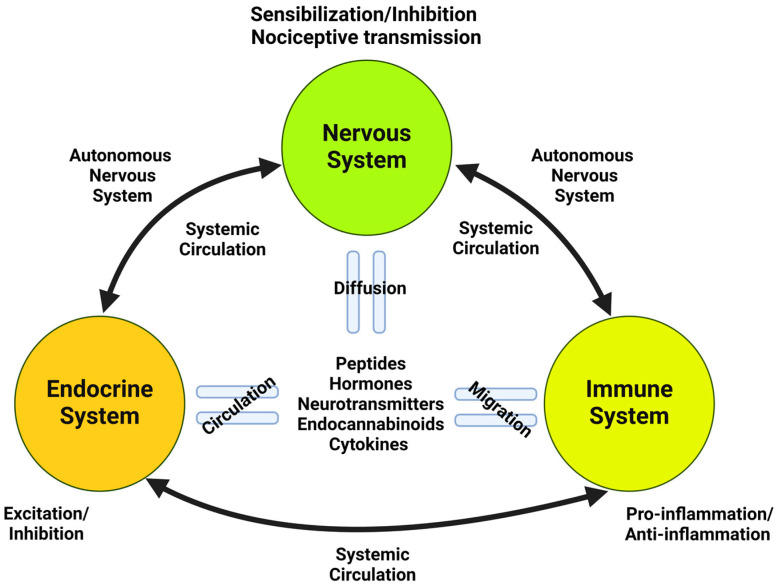
Schematic representation of the connectivity between the nervous, endocrine, and immune systems through the autonomous nervous system and systemic circulation.

Endocannabinoids (eCBs) are endogenous signaling molecules naturally produced by all vertebrate animals, including humans. These lipid mediators are predominantly within the central nervous system (CNS) [70,71,72]. Additionally, eCBs are found in cells of the immune and reproductive systems, highlighting their diverse physiological roles [73,74]. These substances, consisting essentially of fatty acids derived from arachidonic acid (AA) metabolism, are critical regulators of various physiological processes, particularly within the CNS. They act as vital stress response regulators, aiding in adaptation or habituation to stress, guarding against the onset of stress-related illnesses and dysfunctions, and ultimately, promoting survival. They achieve this by interacting with autonomic, endocrine, and immune processes and sensory signaling mechanisms [75,76] (Figure 2). In addition, eCBs have been implicated in many behavioral processes, including memory [77,78], emotional state [79,80], feeding [81], inflammation [82,83], hemodynamic response [84,85], energy metabolism [86,87], pregnancy [88,89], and nociception [90,91,92]. Moreover, they also modulate the proliferation, motility, adhesion, and apoptosis of cells [93,94]. 

Our understanding of the endocannabinoid system (ECS) has grown significantly since the discovery of the first eCBs, anandamide (N-arachidonoylethanolamine, AEA) [15] followed by 2-arachidonoyl glycerol (2-AG) [16], both considered the primary players. These lipid mediators derived from arachidonic acid (AA) have cannabis-like effects. Further exploration has revealed additional eCBs within the brain, including the ether-linked 2-arachidonoyl-glyceryl ether (noladin ether), the AA ethanolamine derivative virodhamine, and N-arachidonoyldopamine (NADA). Notably, NADA acts primarily as a transient receptor potential vanilloid type-1 (TRPV1) agonist but also exhibits some activity at the CB1r. Additionally, structurally related compounds such as N-acylethanolamines (e.g., N-oleoyl ethanolamine (OEA) and N-palmitoyl ethanolamine (PEA)) and 2-oleoylglycerols (e.g., 2-oleoyl-glycerol and 2-linoleoyl-glycerol) are widely distributed in both the CNS and periphery, forming part of the expanded ECS. Nevertheless, their endocannabinoid classification remains contentious due to their lack of affinity for CB1r and CB2r [95,96,97].

eCBs are synthesized on demand, meaning they are not stored pre-formed within cells for later release. Following their release, their biological effects are rapidly terminated by cellular uptake and/or subsequent enzymatic degradation [98]. However, accumulated evidence mainly from pharmacological studies strongly suggests that there must be a maintained cannabinoid tone with a continuous release of endogenous ligands [99]. It is worth mentioning that eCBs are synthesized from membrane precursors, and the degradation products of eCBs serve as precursors for eicosanoids. Consequently, eCB signaling is integrated into a lipid metabolism and signaling network. Therefore, altering the activity of enzymes involved in eCB synthesis and degradation may also affect other lipid signaling systems [100].

Similarly, the distinct distribution patterns of enzymes responsible for eCBs synthesis and degradation throughout the cell and its compartments suggest diverse functional roles for these molecules [101]. Moreover, AEA and 2-AG exhibit distinct pharmacological profiles, interacting with CB1r and CB2r and other receptors like TRPV1 and GABAA [102,103]. Additionally, endogenous peptides known as pepcans or hemopressins can influence biological processes by acting on CB1r and CB2 [104,105,106]. 

#### Involvement of eCBs in the Regulation of Pain

Endocannabinoids (eCBs) modulate pain perception through a dual mechanism. The first involves the activity-dependent phasic release of eCBs triggered by neuronal activity. The second involves a sustained endogenous eCB tone, elevated in pathophysiological conditions like inflammation [99,107].

The diversity of cannabinoid-mediated signaling, the ligand concentration, the presence of other cannabinoid ligand molecules, and the different distributions of metabolic enzymes influence the response to specific eCBs. In this regard, the pathological state and tissue type significantly affect the levels of eCBs and related compounds [108]. These variations likely arise from the disease-specific alterations in enzymes responsible for eCB metabolism. These enzymes exhibit distinct functions, leading to variable effects on the metabolism of different eCBs and related lipids within the same family. Consequently, the levels of fatty acid amide hydrolase (FAAH), cyclooxygenase (COX), and lipoxygenase (LOX) may vary depending on the pathological condition [100]. Molecular studies have demonstrated modulation of the endocannabinoid system (ECS) following spinal cord injury (SCI), with changes observed during both the acute and chronic phases [108,109]. Specifically, AEA is upregulated during the first week after injury. Similarly, alterations in eCBs have been observed in neuropathic pain across various regions of the pain pathways of ascending and descending pain pathways [110,111].

Notably, CB1r is abundantly expressed in neurons and oligodendrocytes, being the AEA/CB1r system critical for neuronal survival [108,109]. Shifting to the chronic injury phase, two to three weeks after injury, there is an increase in 2-AG, a molecule that can activate both CB1r and CB2r. Furthermore, alongside these endocannabinoid changes, the chronic injury phase also witnesses increased CB2 receptor levels in macrophages and astrocyte-like cells [108,112]. CB2r was first considered a peripheral restricted cannabinoid receptor that could be present in the CNS only under certain pathological conditions [113]. However, since the publication of a study identifying the expression of CB2r in neurons of the brainstem of mice, rats, and ferrets under normal physiological conditions [114], much attention has been paid to the functional role it might play, particularly concerning neuroinflammatory processes [22,115].

At present, the diversity of ECS signaling molecules and their interactions with various receptors, together with the signaling complexity of receptor systems, makes the pharmacological intervention of the ECS a challenging task, containing a considerable degree of unpredictability in the outcome of the biological effects in a whole organism.

### 2.3. Synthetic Cannabinoids

Synthetic cannabinoids are molecules developed in laboratories that interact with cannabinoid receptors, thereby achieving a therapeutic effect [116]. Among these, the phytocannabinoids THC and CBD are available as synthetic compounds for a range of indications, as outlined below. Various pharmaceutical products are available in tablets, capsules, and sprays, which can only be obtained via prescription [117].

Some examples of synthetic cannabinoids are the following: (1) nabilone (Cesamet^®^ or Canemes^®^) is a synthetic analog of delta-9-THC with a different molecular structure than THC, which gives it a slightly different interaction with cannabinoid receptors. It is marketed in capsules for oral administration. Cesamet is manufactured by Meda Pharmaceuticals Inc.(Somerset, NJ, USA) and Canemes by AOP Orphan Pharmaceuticals AG (Canonsburg, PA, USA). Its use is approved for the treatment of nausea and vomiting caused by chemotherapy as well as for pain control [118,119,120,121,122]. (2) Dronabinol (Marinol^®^, Adversa^®^, Syndros^®^, and Reduvo^®^) is an oral capsule or oral solution containing a synthetic analog of delta-9-THC prepared in 2.5 mg, 5 mg, or 10 mg. Marinol is produced by AbbVie Inc. (North Chicago, IL, USA) and Syndros by Insys Therapeutics Inc. (Chandler, AZ, USA). Its use has been approved for treating nausea, vomiting, loss of appetite, and weight loss [123,124]. (3) CBD (Epidiolex^®^) is a drug marketed as a viscous oral solution containing CBD as the main active ingredient (100 mg per mL). This medication is indicated for the treatment of seizures in patients with Lennox–Gastaut syndrome, Dravet syndrome, or tuberous sclerosis complex [125,126]. (4) Nabiximol (Sativex^®^) is an oromucosal spray containing approximately a 1:1 combination of THC and CBD extracted from the *Cannabis* plant, delivering 2.7 milligrams of THC and 2.5 milligrams of CBD per dose, manufactured by GW Pharmaceuticals Plc. This oromucosal spray is primarily indicated for managing spasticity associated with multiple sclerosis, particularly in patients who have not responded adequately to other therapies [127,128]. However, there is literature on treating neuropathic pain of different origins [129]. (5) Rimonabant (Acomplia™ and Zimulti™) is a synthetic cannabinoid compound characterized by a CB1r inverse agonism/antagonism mechanism of action and was designed to decrease appetite and promote weight loss in obese patients [130,131]. Despite its effectiveness in achieving the purposes for which it was intended, rimonabant was discontinued, and its sale is currently banned due to its association with depression and suicide attempts.

## 3. Molecular Mechanisms Underlying Analgesic Effects of Cannabinoids

Pain arises from a complex, multi-layered pathway within the nervous system. Sensory information travels from the site of injury through the dorsal horn of the spinal cord and relays to structures of the brainstem and diencephalon, including the thalamus, periaqueductal gray, parabrachial nucleus, reticular formation, amygdala, and hypothalamus, among others [132,133]. This intricate network integrates signals from both tissue damage (nociceptive pain) and dysfunctional brain processing (neuropathic pain) to create the final sensation of pain [134,135,136]. Likewise, the body activates the endocrine and immune systems to counter aversive stimuli and promote healing [137,138,139]. These integrated physiological processes culminate in a defensive biological response to injury [140,141].

Consequently, exposure to a repeated noxious stimulus, such as during tissue damage or exposure to too intense stimuli [142], triggers a neuroinflammatory phenomenon associated with an increased response of nociceptors (peripheral sensitization) [143] and increased excitability of the neurons in the spinal cord (central sensitization) [144,145,146] and in the cortical area (cortical sensitization) [147,148]. This response is mediated by several substances acting through cellular and molecular mechanisms. These mechanisms include the following: (1) significant cellular changes that result in ectopic and/or spontaneous nerve discharges, peripheral, and central hyperexcitability and phenotypic changes in conduction pathways, neurodegeneration, and reorganization of cell morphology; (2) molecular changes, highlighting the accumulation and increased expression of sodium channels in the periphery, increased activity of glutamate receptors, particularly the NMDA receptor, reduced GABAergic activity, changes in calcium penetration into neurons, and increased cytokines, chemotactic factors, growth factors, and ATP; and (3) changes in the structural and functional activity of neurons at the central and peripheral levels as well as neuroimmune interactions, which become more prominent during inflammatory reactions [135,149,150,151,152].

In the case of POP, current research on persistent pain management suggests that it may represent a distinct and common subtype of acute pain, differing from pain arising from antigens, chemical nociception, or neuropathic origin. This distinction in pathophysiology underscores the potential need for tailored treatment approaches for POP compared to other pain conditions. For instance, while spinal N-methyl-D-aspartate (NMDA) receptor antagonists effectively alleviate hypersensitivity in various pain models, they often lack efficacy in managing POP [153]. This evidence shows distinct molecular mechanisms at play in different types of pain. Conversely, intrathecal administration of non-NMDA receptor antagonists [154], NK-1 receptor antagonists [155], and cyclooxygenase-1 inhibitors [156] demonstrates promise for treating POP with minimal effects on nerve injury models.

Similarly, the descending facilitatory pathway originating from the rostral ventromedial medulla, known to contribute to behavioral hypersensitivity in inflammatory and neuropathic pain models, appears irrelevant in postincisional pain [157,158]. Interestingly, glial cell activation emerges as a potential factor in the development and persistence of pain after peripheral nerve injury [159]. This highlights the potential of targeting glial and neuronal–glial interactions for novel pain management strategies [160,161].

**Figure 3:**
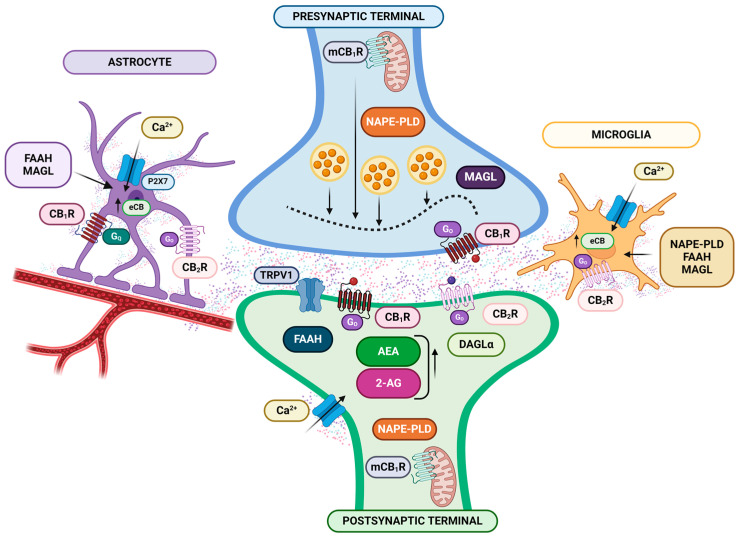
Schematic representation of the main endocannabinoid system components, including the expression of CB1r and CB2r in the CNS. One of the significant properties of Go protein-coupled CB1r/CB2r is that they inhibit the release of a series of transmitters (e.g., glutamate) from the presynaptic terminal. Abbreviations: AEA: anandamide; 2-AG: 2-arachidonylglycerol; CB_1_R/CB_2_R: cannabinoid receptor type 1/type 2; DAGLα: diacylglycerol lipase alpha; eCB: endocannabinoid; FAAH: fatty acid amide hydrolase; Go and Gq: different sets of G proteins; MAGL: monoacylglycerol lipase; mCB1R: mitochondrial CB1 receptor; NAPE-PLD: N-acyl phosphatidylethanolamine phospholipase D; P2 × 7: P2 × 7 purinergic receptor; TRPV1: transient receptor potential cation channel subfamily V, member 1.

In this context, cannabinoids exert their antinociceptive effect through interaction with the ECS, a network of lipid-based signaling molecules that includes two G protein-coupled receptors (CB1r and CB2r), eCBs (AEA and 2-AG) which interact with these receptors [99,162], and two significant enzymes regulating the metabolism of eCBs (FAAH, which predominantly degrades AEA, and monoacylglycerol lipase (MAGL), which predominately degrades 2-AG) [110]. As represented in Figure 3, ECS components are present in neurons, astrocytes, oligodendrocytes, and microglia. CB1 receptors are predominantly localized to the plasma membrane of neurons, with a smaller population residing in the mitochondria (mCB1r). Presynaptic CB1 receptors regulate neurotransmitter release via a retrograde signaling mechanism. An increase in postsynaptic Ca^2+^ levels prompts the synthesis of eCBs within the postsynaptic neuron. Subsequently, these eCBs travel back (retrogradely) to the presynaptic terminal, activating CB1 receptors and inhibiting neurotransmitter release.

Therefore, the ECS is crucial in pain management as a critical modulator of synaptic function within the central nervous system (CNS) [99]. This extends their influence beyond pain perception, regulating various neural functions and behaviors within the immune and endocrine systems [75,76]. In this sense, glial cells influenced by the ECS release a spectrum of signaling molecules (chemokines and cytokines) within the CNS. This bidirectional communication between the nervous and immune systems facilitates adaptation or habituation to stress, protects against stress-induced pathologies, and ultimately promotes survival [163,164,165,166]. Additionally, the ECS exhibits potent antinociceptive and anti-inflammatory activities through interactions with diverse molecular targets, as demonstrated in in vivo studies (see [167,168] for reviews). Moreover, eCBs also significantly regulate hormone production, influencing hypothalamic-releasing factors, pituitary hormones, and peripheral steroidogenesis (see [169] for a review). 

Extensive research is ongoing to elucidate the mechanisms and sites of action responsible for cannabinoid analgesia. CB1r and CB2r are prime candidates, with evidence pointing toward their involvement at the peripheral, spinal, and supraspinal levels [115,170]. Like many G protein-coupled receptors (GPCRs), these receptors exhibit remarkable versatility and adaptability. This is evident in their flexible ligand binding, diverse intracellular signaling pathways, ability to form homodimers and heterodimers, and varied subcellular localization throughout the body [171]. Similarly, evidence suggests that other receptors contribute to ECS signaling [171]. These include orphan G protein-coupled receptors GPR119 [172] and GPR55 [173,174,175,176], as well as peroxisome proliferator-activated receptors (PPARs) [8,175,177,178,179,180,181]. Moreover, cannabinoids can additionally activate ion channels, particularly TRPV1 receptors. The expression of TRPV1 on sensory nerves is known to mediate inflammatory pain, and TRPV1/CB1 receptor co-expression is enhanced in inflamed tissue [182,183]. 

**Figure 4:**
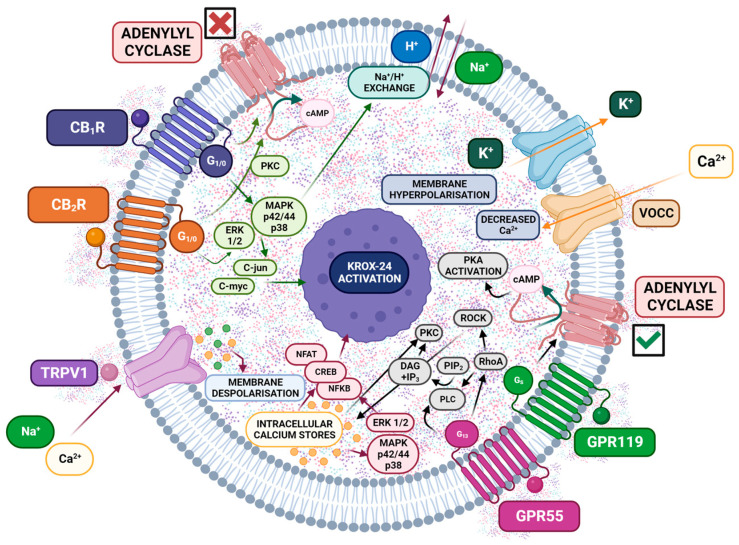
Diagram illustrating the critical signaling pathways triggered by the activation of cannabinoid receptors. CB1r activation triggers Gi/o coupling, leading to inhibition of adenylyl cyclase, modulation of membrane ion channels, and activation of MAPK/ERK and transcription factor signaling pathways. This ultimately results in membrane hyperpolarization. Activation of the CB2 receptor leads to Gi/o coupling, inhibiting adenylyl cyclase and activating MAPK/ERK signaling and transcription factors. Activation of the GPR119 receptor leads to Gs coupling, stimulating adenylyl cyclase, increasing cAMP levels, and activating PKA. Activation of the GPR55 receptor leads to G13 coupling, activating the phospholipase C and RhoA pathway, resulting in increased intracellular calcium levels, activation of MAPK/ERK signaling, and translocation of transcription factors. Activation of the TRPV1 receptor causes a non-selective influx of cations and membrane depolarization. Finally, CB1 and CB2 receptor activation leads to KROX-24 activation through the MAPK/ERK signaling cascade. KROX-24 is a regulatory nuclear transcription factor closely associated with critical biological functions such as stabilizing long-lasting, long-term potentiation, cell differentiation, survival, or death signaling in neuronal cells or regulation of specific neurotransmitters and receptors.

Consequently, activating different cannabinoid receptor types and locations leads to varied responses to noxious stimuli [184]. Thus, metabotropic receptors, e.g., CB1r, CB2r, GPR55, and GPR119, are associated with a slower reaction but with a longer-lasting and more far-reaching action by allowing the opening of different channels for a longer time since second messengers can act in cascade (generating the activation of other proteins and substances). Typical intracellular events mediated by Gi/o proteins coupled with CB1r activation include inhibiting most voltage-dependent calcium channels and increasing potassium conductance [185,186,187,188,189,190]. They also stimulate the mitogen-activated protein kinase (MAPK) pathway to regulate proliferative and differentiative phenomena [72]. Both phenomena contribute to reducing neuronal excitability and suppressing neurotransmitter release. In this sense, activation of the CB1r inhibits the release of GABA or glutamate and neuropeptide by nerve terminals [191,192,193,194]. In the case of the CB2r, the transduction mechanisms coupled to the stimulus of this receptor are similar to those of the CB1r. Therefore, activation of the CB2r leads to inhibitory effects on the adenylate cyclase/AMPc system, as well as stimulation of the mitogen-activated kinase pathway (ERK, JNK, and p38) and the PI3KAkt pathway [195], pathways that are closely related to the processes of cell proliferation and survival and are therefore associated with their modification (Figure 4).

In contrast, interaction with ionotropic receptors such as TRPV1 generates rapid, short-lived responses. This suggests that cannabinoid modulation of inward currents through these receptors (ICRs) could activate sensory neurons, potentially leading to nociception (pain perception) [196,197,198]. However, behavioral studies contradict the potential nociceptive effects suggested by ionotropic receptor activation [196,199,200]. These studies report cannabinoid-induced antihyperalgesia and antinociception, signifying pain reduction at the periphery [168,201,202]. One possible explanation lies in the type of cannabinoid action on these receptors. Unlike full agonists, cannabinoids may only partially activate ICRs, which is insufficient to trigger nociceptor excitation [199,203,204]. Several studies show that cannabinoids evoke slow, small, inward currents and calcium accumulation, potentially falling below the threshold for pain activation [199,203,204]. Moreover, slow depolarization of nociceptor membrane potentials might lead to the inactivation of voltage-gated channels, which inhibits the generation of action potentials [205]. 

Notably, cannabinoids also can modulate the release of mediators involved in pain and inflammation. For instance, activation of CB1 receptors on neuronal presynapses reduces cellular activity and consequently diminishes the release of neurotransmitters like dopamine, noradrenaline, serotonin, GABA, and glutamate retrogradely [206,207,208,209,210]. Additionally, cannabinoids influence other biological systems through the control that second messengers may undergo and even through allosteric changes secondary to the insertion of cannabinoids in the cell membrane [211]. Thus, although classic descriptions of eCBs focus on interactions between the nervous and immune systems, recent research emphasizes their regulatory influence on endocrine function. This extends to various hormonal axes, including those controlling gonadal steroid, growth hormone, prolactin, thyroid hormone, and HPA axis activity [212,213].

Furthermore, although eCBs are known for localized action due to their rapid breakdown [164], the ability of cannabinoids to affect pain perception has supraspinal, spinal, and peripheral components within its general strategy of action of a local modulatory system [214]. Direct evidence for supraspinal cannabinoid antinociception has been substantiated by administration of cannabinoid agonists intracerebroventricularly and/or into encephalic structures at minimal doses [215,216,217,218], administration of supraspinally administered cannabinoid antagonists inducing pain [219,220], and electrical stimulation of the rat periaqueductal gray (PAG), as well as formalin injection into the hind paw and increased AEA release in the PAG as determined by microdialysis coupled to liquid chromatography/mass spectrometry [221], respectively. On the other hand, evidence of spinal cannabinoid antinociceptive effects has been obtained through behavioral, neurochemical, and electrophysiological studies using spinal cannabinoid agonists [222,223]. In this case, cannabinoids can act on spinal CB1rs to inhibit capsaicin-sensitive fibers in the dorsal horn and reduce the firing of wide dynamic range (WDR) neurons in response to noxious stimuli [222,224]. Additionally, activation of the spinal CB1r can decrease NMDA receptor activation by potentially inhibiting glutamate release into the spinal cord [225], and activation of CB2r suppresses activity in spinal nociceptive neurons, particularly under conditions of sensitization, and regulates the immune response, favoring the neuroprotective actions of neuroglia [226,227].

Furthermore, cannabinoids may modulate spinal noradrenergic and opioid systems [228,229]. Concerning the peripheral component, the antinociceptive action of CBr has been demonstrated in different pain models by peripherally administering CB1r [230] and CB2r [231,232,233] agonists. Likewise, it has been confirmed that endocannabinoid substances such as AEA also activate TRPV1 receptors [234]. For a more detailed review of the mechanisms involved in the analgesic effects of cannabinoids, please see [235,236].

In summary, the interaction of CBr agonists (either endogenous or exogenous) leads to a reduction in neuronal activity secondary to inhibition of bioelectrical activity by lowering the intracellular level of second messengers and loss of the ability to release their specific neurotransmitters (whatever they activate or inhibit). The cellular consequences are short-term modification of the permeability of membrane ion channels (mainly for K^+^ and Ca^2+^ [237,238]), decreasing neuronal excitability and long-term changes in gene expression that result in phenomena such as brain plasticity, dependence, and transformation of an acute response into a long-term adaptation or memory [189,239,240,241,242]. The overall result is decreased pain perception by modulating the nociceptive impulse at different levels and activating a descending inhibitory system acting on the spinal cord [92,243,244,245]. Multiple factors, such as the diversity of cannabinoid-mediated signaling [246,247,248,249,250], the ligand concentration [247,251,252], the presence of other cannabinoid ligand molecules [252], the exact localization of the cannabinoid receptors [184], and the different distributions of metabolic enzymes influence [110,253] the response to a specific cannabinoid.

## 4. Why Consider the Therapeutic Potential of Cannabinoids in Postoperative Pain Relief?

### 4.1. Activation of the ECS after Surgery

Surgical procedures invariably induce peripheral tissue trauma, initiating a well-orchestrated nociceptive response. The specific tissues implicated vary based on the surgical approach, encompassing skin, fascia, muscle, vasculature, viscera, and potentially neural structures. This damage directly activates nociceptive nerve fibers, particularly unmyelinated C-fibers, contributing to postsurgical pain’s dull, aching quality. Furthermore, tissue injury triggers a robust inflammatory response, a coordinated endeavor involving diverse cell types and releasing many mediators. These mediators are essential to clearing cellular debris, combating potential pathogens, and facilitating wound healing through scar tissue formation. However, the inflammatory process can also contribute to pain by sensitizing nearby nociceptors [254].

A significant challenge in POP arises from iatrogenic nerve injury. Specific surgical interventions, such as limb amputation or inguinal hernia repair, carry a higher risk of peripheral nerve damage [255]. This nerve injury can lead to the development of neuropathic pain [256], a chronic pain state characterized by burning, electrical, or dysesthetic sensations. Neuropathic pain is a prevalent condition, affecting at least 8% of individuals with chronic pain and significantly contributing to chronic postoperative pain (CPOP) [257,258]. It is noteworthy, however, that not all nerve injuries culminate in chronic pain; for instance, only approximately 80% of amputees develop CPOP [149,259].

In this context, preclinical POP studies have shown evidence of a crucial role for the ECS in resolving pain after surgery and preventing its transition into a chronic state by limiting pro-inflammatory responses within spinal cord glial cells [18,19]. Targeting eCBs offers several advantages: (1) localized and on-demand synthesis whereby eCBs are produced at the site of action, minimizing side effects associated with widespread cannabinoid receptor activation [247]; (2) endogenous production whereby the body naturally produces eCBs and the enzymes responsible for its breakdown, resulting in a shorter half-life and potentially lower toxicity compared to synthetic drug; and (3) synergy with existing treatments whereby the ECS can enhance the pain-relieving effects of common nonsteroidal anti-inflammatory drugs (NSAIDs), potentially improving pain management strategies [260]. 

However, despite evidence supporting eCBs’ role in pain relief [19,261,262,263,264] and the effectiveness of inhibiting FAAH and MAGL (enzymes degrading eCBs) in reducing pain [265], research also reveals complexities. Studies have shown increases and decreases in eCB levels depending on the pain-transmitting tissues analyzed [266,267]. Additionally, diverse studies suggest that blocking cannabinoids CB1r and CB2r can produce pain-relieving and anti-inflammatory effects [268,269,270,271]. This indicates that endocannabinoids can act as pro- and anti-inflammatory mediators [270,272], potentially making ECS activation detrimental in certain situations.

The current understanding suggests that the initial eCBs response to acute pain helps restore balance within the body, with precise timing and location being crucial. However, chronic pain states can dysregulate this system, leading to prolonged or inappropriate endocannabinoid activity. This complexity makes predicting the effectiveness of ECS-targeting drugs challenging for specific conditions.

At the clinical level, limited research exists. One study measured various endocannabinoid mediators in plasma, cerebrospinal fluid, and synovial fluid, correlating them with pain levels and opioid use after surgery [273]. The study found a significant correlation between synovial and cerebrospinal fluid 2-AG levels and both pain scores and opioid consumption. However, the potential confounding factor of pre-existing osteoarthritis and chronic pain in some participants needs to be addressed in future studies.

### 4.2. Cannabinoids Themselves Possess Analgesic Properties 

The link between cannabinoid receptors and pain pathways has involved animal studies assessing cannabinoid antinociception [92,274]. Small-molecule CB1r and CB2r agonists and FAAH inhibitors were the most frequently evaluated. Preclinical data obtained with cannabinoid agonists after systemic [230,275] and/or perimedullary [225,230,276] administration in pain models of excess nociceptive, visceral, and neuropathic pain models [214,274,277] concluded that the antinociceptive effect is similar to opioids [278,279,280,281] and that it varies according to the substance chosen, the dose administered (a significant dose-dependent correlation exists between the administered cannabinoid dosage and the degree of antinociception observed) [282,283], and the route of administration (the doses required after administration by the perimedullary route are much lower and the duration of the effects longer than those used to achieve the same result by non-perimedullary routes) [229,284]. They also highlighted the greater antinociceptive power in inflammatory and neuropathic pain models [285,286,287,288]. In all cases, cannabinoids primarily act by inhibiting or releasing a series of modulators from neurons and/or non-neuronal tissues. This targeted action in inflamed tissues, where these modulators are present in relatively high amounts, is hypothesized to lead to a faster and more intense antinociceptive effect [83,277].

Furthermore, cannabinoids can act on various antinociceptive mechanisms, including the modulation of enzymes responsible for generating and releasing inflammatory agents and enhancing the abundance and diversity of cannabinoid receptors [274]. Regarding efficacy in neuropathic pain [289,290], it is relevant to highlight that the central cannabinoid CB2r is required for an anti-allodynic effect [22]. Cannabinoids also activate and desensitize another significant player in neuropathic pain (i.e., TRPV1) [291,292,293]. They reduce microglia and astrocyte activation as a mechanism related to the onset and maintenance of hypersensitivity in neuropathic pain [18,19].

However, human studies have yielded partially supportive findings from animal research regarding the use of cannabinoids for pain management [294,295]. Despite a long history of cannabis use for pain management [296], clinical trials have revealed limited efficacy for acute pain and even potential enhancement of specific pain responses [74,274,297,298,299,300,301,302]. Conversely, moderate-quality evidence suggests a small effect for chronic, non-cancer-related pain management with cannabinoids up to 6 months, potentially due to their influence on neuroplastic changes [303,304,305,306,307]. Therefore, based on the available literature, it is challenging to recommend cannabinoids as a general alternative to the currently marketed analgesic for pain management [302,308].

In this scenario, the complex nature of POP demands the development of more effective and better-tolerated therapeutic approaches. These options should target nociceptive and neuropathic pain pathways to relieve pain sensitization [140,309]. Ultimately, this could mitigate the reliance on opioid medications during POP and minimize the risk of long-term pain complications [11,310,311,312,313,314]. It is worth noting that the activation of the μ-opioid receptor is linked to numerous adverse effects, including respiratory depression [315,316], postoperative nausea and/or vomiting [317,318], constipation [319,320,321], urinary retention [318,322], mental clouding and somnolence [323,324], tolerance [325,326,327,328,329,330], hyperalgesia [313,331,332,333,334], dependence, and addiction [335,336,337,338,339]. Among these, a pivotal strategy is multimodal analgesia [340,341]. This involves combining various medications with different mechanisms of action to address pain from multiple angles. The aim is to minimize side effects from individual drugs and target different pain receptors for optimal pain control and improved recovery. Prioritizing non-opioid medications as the initial treatment for POP offers a safer and potentially more effective approach. These medications often have a wider therapeutic window, implying the dosage range for effective pain relief with minimal side effects.

To date, cannabinoids are emerging as promising candidates to replace or reduce reliance on other long-term pain medications. This potential stems from their ability to modulate the ECS, which in turn regulates the release of various neurotransmitters critical for pain perception, such as glutamate, GABA, serotonin, acetylcholine, dopamine, and norepinephrine [342,343,344]. However, research on the synergistic effects between existing analgesics and cannabinoid receptor agonists is limited. Nevertheless, several combinations promise enhanced pain management with reduced side effects. These include combinations with opioids, nonsteroidal anti-inflammatory drugs (NSAIDs), paracetamols, local anesthetics, and α2-adrenergic receptor agonists.

### 4.3. Opioids—Cannabinoids

The potential of cannabinoids to decrease opioid dosages and prolong the duration of adequate analgesia justifies further research. This is particularly intriguing considering their shared distribution within the descending pain inhibitory pathway and potential complementary cellular mechanisms of action compared to opioids [345] interacting synergistically in attenuating pain [346]. Thus, eCBs are produced and released within active neural circuits, where they play a critical role in mediating an adaptive response to mitigate pain and inflammation following injury and stress [247,347]. Pieces of evidence to date indicate that an ECS physiological tone mediates this regulation. In addition, ECS tone changes under pathophysiological situations such as inflammation, acting synergistically with the opioid system through different mechanisms [348,349]. A key consideration is the direct interaction between CB1r and mu-opioid receptors as functional heterodimers within the same neuron, as demonstrated when co-expressed [350]. Furthermore, cannabinoid administration can stimulate the synthesis and release of endogenous opioid peptides in the central nervous system and periphery [351]. 

While these properties imply a potential synergistic interaction between opioids and cannabinoids, the situation is not entirely straightforward due to the complexities of cannabinoid pharmacology. For example, the activation level of cannabinoid receptors (both basal and stimulated) by different agonists significantly influences the observed effects [352,353]. Moreover, partial agonists such as anandamide can act antagonistically in the presence of more efficacious agonists [354].

At present, preclinical studies support opioid sparing when co-administered systemically [355,356,357,358,359], intradurally, and/or intracerebroventricularly [229,360], or in a combination of routes [361,362]. 

However, clinical trials investigating nabilone and dronabinol in acute POP revealed no discernible benefits regarding opioid dose requirements or analgesic outcomes [363,364]. 

### 4.4. NSAIDs—Cannabinoids

NSAIDs primarily exert their antinociceptive effect by inhibiting the production of prostaglandins through COX enzyme blockade. COX-1 is constitutively expressed, while COX-2 is induced explicitly during inflammation. Notably, evidence suggests that NSAIDs may have additional pain-relieving mechanisms [365,366,367,368,369]. This has sparked research into potential interactions between cannabinoid agonists and NSAIDs for managing pain and inflammation. This interest stems from the convergence of pathways between endogenous cannabinoid receptor ligands and prostaglandins (molecules derived from arachidonic acid) [370]. Furthermore, evidence from cannabis signaling and the accumulation of arachidonic acid in brain slices exposed to cannabis derivatives supports this exploration [371].

Preclinical research suggests that eCBs and synthetic cannabinoids exhibit synergistic antinociceptive effects when combined with common NSAIDs [372,373,374,375,376]. This finding holds promise for the development of novel pain management strategies. However, further investigation is necessary to determine optimal dosing, safety profiles, and administration protocols for these combinations.

To our knowledge, there have been no clinical studies evaluating the analgesic efficacy of the combination of cannabinoids with NSAIDs.

### 4.5. Paracetamol—Cannabinoids

Several studies have revealed a surprising link between acetaminophen, the most commonly used pain medication, and the endocannabinoid system [377,378]. Research shows that blocking CB1r reduces the antinociceptive effects of paracetamol [379,380,381]. This effect is also observed in CB1 receptor-deficient (CB1–/–) mice [377,378] and those lacking FAAH (FAAH–/– mice) [382]. The link to the ECS becomes more evident when considering that acetaminophen can transform into N-arachidonoyl-phenolamine (AM404). This bioactive compound activates cannabinoid receptors and prevents the breakdown of natural pain-relieving endocannabinoids [383].

To our knowledge, there have been no clinical studies evaluating the analgesic efficacy of the combination of cannabinoids with paracetamol.

### 4.6. Local Anesthetics—Cannabinoids

Because endocannabinoid receptors are present in peripheral sensory afferents and spinal cord neurons [384,385,386,387], another strategy could be to develop synergistic interactions between cannabinoids and local anesthetics. Although the mechanisms for cannabinoid-induced antinociception are still unclear, there is literature that confirms a synergistic effect in experimental models [388].

To our knowledge, no clinical studies have evaluated the analgesic efficacy of combining cannabinoids with local anesthetics.

### 4.7. α2-Adrenergic Receptors—Cannabinoids

Alpha-2 agonists offer significant pain relief with the potential to decrease opioid consumption by attenuating nociceptive signaling throughout the nervous system, from peripheral sites to the brain [389,390,391]. Interestingly, they share similarities with CB1r agonists. Both belong to the G protein-coupled receptor family and can work together through various signaling pathways or by inhibiting adenylyl cyclase, resulting in decreased levels of cAMP and reduced activity of Ca^2+^ and K^+^ channels [392]. Synergistic analgesia can be achieved when a CB1r agonist is combined with an alpha-2 receptor agonist (such as clonidine, tizanidine, or guanfacine). Additionally, modulating the noradrenergic system, which regulates the activity of the hypothalamic–pituitary–adrenal axis (HPA), can be significant in treating anxiety and opioid withdrawal [393].

To our knowledge, no clinical studies have evaluated the analgesic efficacy of combining cannabinoids with alpha-2 agonists.

## 5. Cautions and Limitations of Using Cannabinoids for Postoperative Pain Relief

### 5.1. Which Product Is Suitable for Postoperative Pain Control?

The substantial heterogeneity among studies examining cannabinoids for POP pain management poses a significant challenge. These studies often utilize different cannabinoid medications with diverse mechanisms of action and varying activity levels of CB1r and CB2r, making it challenging to identify the most effective option. For example, THC [297,364,394], levonantradol [395,396], nabilone [363,397], and dronabinol [398] were used in POP. Additionally, CBD products have been employed in POP studies [399,400,401]. Two research articles used investigational compounds under development [402,403]. In this regard, it is not clear whether CBD is a better or worse analgesic than THC [404]. Nonetheless, patients typically perceive marijuana as potentially at least somewhat effective for pain management and are often open to using cannabinoid compounds for this purpose if recommended by a physician [405]. It is also known that nausea responds to THC, while anxiety responds better to CBD [406].

To date, unlike recreational users who prioritize psychoactive effects, medical cannabis patients often seek CBD-rich chemovars (strains) with minimal THC. These chemovars offer the potential for greater symptom control, improved functionality, and enhanced quality of life while minimizing unwanted side effects [407].

In the absence of evidence or clinical guidelines based on rigorous studies, it is advisable to use a cannabinoid product by assessing the effects obtained. A recommended strategy for cannabis initiation is “start low, go slow, and stay low”.

### 5.2. Pharmacokinetic Considerations and Routes of Administration of Cannabinoids in Postoperative Pain Relief

Pharmacokinetics, which involves a drug’s absorption, distribution, metabolism, and elimination, significantly influences its onset and duration of action. These are especially important for cannabinoids. Factors such as the route of administration—currently, cannabinoids have been marketed for systemic and topical use (Table 1)—and pharmacokinetic profile jointly dictate the clinical effects [408,409,410,411].

**Absorption.** Due to their limited aqueous solubility and lipophilic character, cannabinoids display significant variability in their effects depending on the chosen route of administration. 

**Pulmonary route:** Inhaling cannabinoids brings on effects quickly, within 15 min, then levels off for 2–4 h before slowly wearing off. Their bioavailability varies considerably (between 10% and 85%), owing to various factors such as individual differences in inhalation techniques (number of puffs, duration and interval of puffs, breath hold time, and depth of inhalation), the device used, the size of inhaled particles, the temperature of the vaporizer, and the site of deposition within the respiratory system [410,411,412]. 

**Oral route:** THC and CBD formulations have a low bioavailability of around 6% due to their lipophilic structures, variable gut absorption, and extensive hepatic first-pass metabolism. Plasma concentrations for therapeutic effects remain within range for 2 to 6 h [409,411,413]. However, blood concentrations only reach 25–30% of those achieved through smoking the same dose. This is because first-pass metabolism by the liver reduces THC reaching circulation, although the resulting metabolite 11-hydroxy delta-9THC retains some psychoactive effects. The onset of the effect is delayed (0.5–2 h) and may be prolonged by continued slow absorption from the gut [414]. Notably, cannabinoids are best absorbed with fat, oils, or polar solvents like ethanol. Newer technologies, such as using nano- or ionized particles or incorporating omega fats into carrier oils, suggest a potential for increased absorption [415]. Additionally, the design of new water-soluble cannabinoid agonists opens up new possibilities for improved bioavailability [416]. 

**Mucosal-related pathways:** The sublingual and buccal regions of the oral cavity are lined with a non-keratinized, stratified, squamous epithelium. This specialized tissue is a selective barrier, allowing certain substances to pass through [417]. In this case, the formulation of THC and CBD (Sativex^®^) facilitates rapid absorptions and bypasses hepatic first-pass metabolism, resulting in higher plasma levels achieved through oral administration but lower than through inhalation administration [418]. However, sublingual and buccal routes have some drawbacks compared to oral administration. These include the following: (1) a shorter duration of action whereby the pain relief effects wear off quicker; (2) frequent dosing whereby maintaining stable pain control requires repeated administrations and this can increase the risk of side effects; and (3) potential for adverse reactions whereby rapid administration can lead to high drug concentrations in the bloodstream, raising the chance of severe reactions. Advances in nanoparticulate drug delivery represent a line of research aimed at enhancing the retention and absorption of drugs in the buccal and sublingual regions [419]. 

**Skin-related pathways:** By bypassing first-pass metabolism, transdermal cannabinoids can potentially offer a more consistent and controlled release of cannabinoids into the body compared to edibles [420]. However, their water-insoluble nature requires permeation enhancers to ensure they reach the bloodstream effectively [421,422]. Studies indicate that CBD exhibits ten times greater permeability in transcutaneous administration compared to THC. This finding suggests that CBD possesses a more polar structure than THC [410,423,424].

Intravenous route: Intravenous administration of cannabinoids presents a unique challenge due to their poor water solubility. Nonetheless, it remains the most reliable method for the administration of synthetic cannabinoids. This route bypasses first-pass metabolism, ensuring minimal variability in plasma concentrations and consistent results across patients. The resulting plasma profile following an intravenous dose closely mirrors that observed after inhalation. However, rapid redistribution within the body leads to a swift decline in plasma levels. Subsequently, drug metabolism contributes to a slower, sustained decrease in concentration [425]. It is important to note that the existing literature regarding the use of intravenous cannabinoids for postoperative pain management remains limited. Additionally, the research conducted thus far has primarily focused on the effects of tetrahydrocannabinol (THC) [426].

**Distribution**. Upon absorption, THC and other cannabinoids rapidly distribute to various tissues at rates influenced by blood flow [214,409,427]. Because they are extremely lipid-soluble, cannabinoids tend to accumulate in adipose tissues, reaching peak concentrations within 4–5 days. Subsequently, they undergo slow release into other body compartments, including the brain. Due to sequestration in fat, THC has a tissue elimination half-life of approximately 7 days, with complete elimination of a single dose potentially taking up to 30 days [428]. This accumulation phenomenon suggests that with repeated dosage, cannabinoids can persist in the body and continue to reach the brain. In the brain, THC and other cannabinoids exhibit differential distribution, with high concentrations observed in the neocortical, limbic, sensory, and motor areas. Notably, the volumes of distribution (Vd) for CBD and THC are notably high. Specifically, the volume of distribution at beta phase (Vdβ) is approximately 32 L/kg following intravenous administration for CBD [429], and the volume of distribution at steady state (Vdss) is approximately 3.4 L/kg following inhaled administration for THC [411].

**Metabolism**. The metabolism of THC primarily occurs in the liver, predominantly through cytochrome P450 (CYP450) isozymes such as CYP2C9, CYP2C19, and CYP3A4. THC is primarily metabolized into 11-hydroxy-THC (11-OH-THC) and 11-carboxy-THC (11-COOH-THC), which undergo glucuronidation and are subsequently excreted in the feces and urine [410]. Additionally, metabolism occurs in extrahepatic tissues expressing CYP450, such as the small intestine and brain [411]. It is worth noting that the metabolite 11-OH-THC is found in higher quantities in the brain compared to the unmetabolized THC compound, suggesting a potential role for 11-OH-THC in the effects experienced with THC [424,430,431]. The increased uptake of 11-OHTHC in the brain may be attributed to its lower plasma protein binding or the hydroxylated metabolite’s ability to pass through the blood–brain barrier [411]. In the case of CBD, it undergoes extensive hepatic metabolism, primarily by the cytochrome P450 (CYP) isozymes CYP2C19 and CYP3A4. Additional contribution comes from CYP1A1, CYP1A2, CYP2C9, and CYP2D6 [432]. Following hydroxylation to 7-OH-CBD, further hepatic metabolism primarily leads to fecal excretion, with a minor contribution to the urinary excretion of these metabolites. However, the pharmacological activity of CBD metabolites in humans remains largely unknown [433].

**Elimination**. The elimination half-life of THC demonstrates biphasic characteristics. A population pharmacokinetic model estimates a rapid initial half-life of approximately 6 min, followed by a slower terminal half-life of around 22 h [434]. This extended terminal phase is attributed to equilibration between THC stored in lipid compartments and its release back into the bloodstream [435]. Heavy cannabis users display an even longer terminal half-life due to the slow redistribution of THC from deep fatty tissues [436].

Consequently, blood THC concentrations exceeding 1 μg/L may persist for more than 24 h after their last use in heavy users [436,437]. Conversely, CBD also exhibits a prolonged terminal elimination half-life. Following intravenous administration, the average half-life is 24 ± 6 h, while inhalation results in a slightly longer value of 31 ± 4 h [429]. Notably, repeated daily oral administration of CBD leads to a significantly extended half-life, ranging from 2 to 5 days.

### 5.3. Potential Interactions of Cannabinoids in Postoperative Pain Relief

The metabolism of cannabinoids, particularly THC breakdown by cytochrome P450 (CYP) enzymes [438], suggests potential interactions with various drug classes [439,440,441]. While clinically significant interactions are rare [442], caution is advised, especially when combining cannabinoids with other central nervous system depressants (increased sedation), serotonin reuptake inhibitors (SSRIs)/antidepressants, sympathomimetics (potential for elevated heart rate and blood pressure), or pain medications [443,444,445].

Existing evidence has not demonstrated toxicity or loss of effect of concomitant medications, although such outcomes are theoretically possible. One exception is the interaction between high-dose CBD and clobazam, where elevated levels of a sedative metabolite, N-desmethyl clobazam, necessitate a dose reduction for that drug. Furthermore, the accumulating literature highlights the interaction of CBD with various catalytic activities of cytochrome P450 isoenzymes, demonstrating its potency as an inhibitor of CYP2C19 [446], CYP2D6 [447], or CYP3A4 [448], among others. Thus, it is crucial to consider potential interactions with other concomitant drugs metabolized by these isoenzymes [449].

### 5.4. Acute Adverse Effects of Cannabinoids 

Our understanding of the effects of cannabinoid agonists in humans is predominantly derived from two sources: clinical observations and anecdotal reports from individuals consuming marijuana, as evidenced in Table 2. The pharmacokinetic profile, particularly the time course of action, exhibits significant variability depending on the administered dose and route. For instance, oral administration leads to a slower onset (30 min to 1 h) with longer-lasting effects (approximately 6 h) than inhalation or oral transmucosal routes. These latter routes offer a rapid onset with potent effects, but the duration is shorter. However, once established, the qualitative nature of the effects often displays a similar pattern across individuals [450]. 

Within the context of POP, the most commonly encountered acute adverse effects of cannabinoids are primarily attributed to their interactions with the central nervous system (CNS). Typically, the consumption of cannabinoids induces in humans an initial feeling of euphoria, well-being, and happiness, followed by a state of drowsiness. During this initial phase, one experiences excitation, dissociation of ideas, increased and distortion of extrasensory perception (increased visual and auditory perception), spatiotemporal errors of appreciation, alterations of emotions, and in some cases, fixed ideas, illusions, irresistible impulses, and hallucinations [428,451]. Other mental and behavioral effects observed in this phase are alterations in memory for recent events [20,452,453], alterations in motor coordination (e.g., driving vehicles), and other psychomotor abilities, difficulties in concentration, especially in complex tasks requiring divided attention, stuporous states (“hanging”), slowing of reactions, decreases in mental activity, and impairments in peripheral vision [454,455]. It is worth mentioning that the effects vary from one individual to another depending on the dose (they increase at higher doses), route of administration, individual vulnerability (personality, expectation, experience of the consumer), as well as the circumstances of consumption. In all cases, they are easily quantifiable, measurable over a few hours (generally no more than 4–6 h) [455,456], and difficult to correlate with plasma levels [457,458]. Dysphoric reactions such as panic and acute anxiety attacks, unpleasant somatic sensations, and paranoid feelings are dose-dependent and occur mostly during initial contact with cannabinoids or individuals with a history of psychosis [459].

Furthermore, their hemodynamic and digestive effects are other undesirable effects to consider during POP. Cannabinoids generally exhibit vasodilatory reflex properties when acting through CB1r [460,461]. This response is multifaceted and may involve three phases: vagal-mediated hypotension (Phase I), followed by a compensatory increase in blood pressure (Phase II), leading to prolonged hypotensive effects (Phase III) [462]. The most consistent cardiovascular effects of both marijuana smoking and i.v. administration of delta-9-THC are peripheral vasodilation and tachycardia (compared with bradycardia in animals), occurring within minutes to a quarter of an hour and lasting up to 3 h [451,463,464,465]. This increase in heart rate may elevate cardiac output and oxygen demand [466]. Blocking drugs can be used to mitigate this effect [467]. Additionally, inhibiting acetylcholine release from the autonomic nervous system fibers after interaction with intestinal CB1r should be considered a cause of intestinal ileus [468]. 

Nevertheless, cannabinoid receptor agonists offer a promising alternative for pain management compared to current medications. Opioids, while effective, can lead to life-threatening complications and contribute to the opioid crisis [10,469]. NSAIDs, such as ibuprofen and diclofenac, although widely used, can induce cardiovascular toxicity through mechanisms like prostaglandin inhibition. In contrast, the acute toxicity of cannabinoids is very low [67]. The dose of THC required to produce 50% mortality in rodents is extremely high compared with other commonly used drugs [470]. 

### 5.5. Patients with a History of Cannabinoid Use

One of the premises of treating POP is the individualization of the guidelines to achieving a satisfactory result and acceptable side effects. In this sense, taking a series of precautions in special risk groups is necessary to reduce undesirable effects. 

Studies indicate that 10–20% of individuals aged 18–25 years may use cannabis weekly or more frequently. Due to its slow elimination from the body, these cannabinoids may persist in the tissues for weeks, potentially interacting with various anesthetic agents and affecting their efficacy [471,472]. Additionally, cannabis use may be associated with higher pain scores and poorer quality of sleep in the early postoperative period [410,412]. Therefore, a systematic preoperative inquiry regarding cannabis use is highly recommended. In cases of recent cannabis use, postponing elective surgery is advisable to minimize potential complications [471,472].

It is noteworthy that cannabinoids significantly enhance the hypnotic and sedative effects of CNS depressants commonly used in general anesthesia (barbiturates, opiates, benzodiazepines)—see above. This can lead to excessive sedation and potential respiratory depression. Additionally, cannabis use can also increase the risk of respiratory complications during general anesthesia. Smoking cannabis irritates the upper airway, causing inflammation (oropharyngitis) and swelling of the uvula (uvular edema) [473]. This swelling can obstruct the airway, especially during breathing tube insertion. In rare cases, cannabis use may contribute to isolated uvulitis, presenting with upper airway pain, fever, hypersalivation, dyspnea, and respiratory distress [474,475]. Furthermore, cannabis use has been linked to laryngospasm [476].

On the other hand, hemodynamic effects are also a concern. Low or moderate doses may cause an increase in sympathetic activity, leading to a faster heart rate (tachycardia) and increased cardiac output. Conversely, high doses can suppress sympathetic activity and stimulate parasympathetic activity, resulting in a slower heart rate (bradycardia) and low blood pressure (hypotension). Cannabis-induced hypotension usually responds well to intravenous fluids [477]. 

In cases of acute cannabis consumption, it is advisable to avoid medications likely to increase heart rate, such as ketamine, atropine, or epinephrine [478]. This is because cannabis can cause pronounced catecholamine release, potentially leading to tachycardia. Conversely, chronic use may cause catecholamine depletion, requiring lower anesthetic doses [471,472].

### 5.6. Contraindications 

Due to potential risks for fetal and neonatal health, including long-term neurodevelopmental effects, cannabis use is contraindicated during pregnancy and lactation [479,480,481]. Similarly, it is contraindicated in psychosis (except for CBD-predominant preparations) [482]. Cannabis should be used with caution in patients with conditions like unstable angina due to tachycardia and possible hypotension from THC, but it does not produce QTc issues [483]. The use of cannabis in children and teens requires further investigation due to potential impacts on cognitive development and academic performance [484]. Similarly, more research is needed to understand its role in addiction and dependency. Smoking cannabis should be avoided for chronic obstructive pulmonary disease (COPD) and asthma.

## 6. Clinical Trials Evaluating Cannabinoids for Postoperative Pain

Eight clinical trials investigating cannabinoids for POP have been conducted, involving a total of 924 patients and utilizing six different cannabinoid compounds, primarily THC or its analogs (e.g., dronabinol) [297,364,394,395,396,397,402,403] (Table 3). Overall, the trials predominantly reported negative findings, with only two studies demonstrating modest (e.g., slight reduction in pain scores) benefits [394,396]. Notably, six out of eight studies administered a single dose of cannabinoids, while in two other studies, administration was extended for 24 and 36 h, respectively.

These findings contradict some systematic reviews suggesting a potential role for cannabinoids in managing pain beyond acute scenarios [46,485]. Meta-analyses evaluating the analgesic efficacy of cannabinoids for acute POP [486,487] concluded that cannabinoids are not ideal for POP due to the following: (1) limited efficacy in that studies show limited pain reduction [394,396] or no effect [297] and (2) potential for hyperalgesia whereby high doses may even worsen pain [397]. To evaluate these findings, the best available qualitative evidence indicates no disparities in cumulative opioid consumption and no variances in the severity of rest pain at 24 h postoperatively.

Cannabinoids are generally well tolerated, with most adverse effects being mild to moderate [486,487]. Common side effects include blurred vision, hypotension, dizziness, drowsiness, dry mouth, hallucination, headache, and nausea. In fact, in five of eight studies [297,364,396,397,402], cannabinoids showed more frequent or severe adverse effects than the placebo for specific events or periods. Nevertheless, analyzing the adverse effect profile of cannabinoids is challenging due to variations in reporting and defining adverse effects among studies. Moreover, some studies failed to assess or report the statistical significance of group differences. For instance, one study noted that patients receiving a placebo were more likely to report postoperative nausea and vomiting compared to those receiving dronabinol, but the statistical significance of this finding was not provided [364]. Additionally, there is an observed increase in hypotension during the postoperative period, posing a risk factor for cerebrovascular disease [488]. A recent randomized controlled trial evaluating the efficacy of intravenous THC in preventing postoperative nausea and vomiting did not recommend its use due to an unacceptable side effect profile and limited efficacy [489].

It is worth noting that despite promising results in animal models, CBD appears ineffective for POP management. Three recent studies explored its therapeutic potential. In the first study, topical CBD administered to patients who had undergone total knee arthroplasty as a supplement to a standardized multimodal analgesic protocol did not reduce pain or opioid consumption [400]. In the second study, buccally absorbed CBD in arthroscopic rotator cuff repair patients showed a suitable safety profile and held promise in reducing pain in the immediate perioperative period [490]. However, a follow-up study indicated that CBD was not able to improve pain scores, patient satisfaction with pain control, or postoperative opioid consumption [399]. Finally, CBD was ineffective in reducing discomfort or opioid usage in patients undergoing ureteroscopy with stent placement for urinary stone disease [401]. 

The Clinical Trial as Proof of Principle of Analgesic Efficacy of Cannabinoids on Postoperative Pain (CANPOP) clinical trial, funded by the Medical Research Council, evaluated the analgesic efficacy of standardized cannabis plant extract (Cannador) administered by p.o. one hour before the intervention in patients with POP (tonsillectomized and patients undergoing abdominal surgery). This clinical study reported significant dose-related improvements in rescue analgesia requirements [394].

In conclusion, the use of cannabinoids for POP presents a mixed picture. While some studies show promise, the evidence suggests limited efficacy and potential side effects.

## 7. Possible Explanations for the Lack of Analgesic Efficacy in Postoperative Pain Relief

Unlike chronic pain, which is often dynamic and neuropathic, POP is acute, localized, and primarily driven by nociceptive mechanisms [491,492]. This difference in pain type might explain the limited effectiveness of cannabinoids for POP compared to chronic pain. Chronic pain is associated with complex changes in the ECS, including upregulation of cannabinoid receptors, altered receptor function, changes in eCB formation or release, and interactions with other pain mediators [393,493]. Supporting this hypothesis, are studies on healthy volunteers, suggesting limited effectiveness of cannabinoids for acute nociceptive pain [298].

Additional factors contributing to the lack of desired results are outlined in Table 4. Notably, the clinical dosage is constrained by the onset of THC-related side effects, being mainly psychotropic [464,494]. Furthermore, an analysis of studies revealed significant disparities in dosing regimens and administration patterns. Timing of administration varied across studies, with some administering cannabinoids preoperatively [363,396,402,403], postoperatively [297,394,395,398], and a few in both settings [412,495]. Moreover, a diverse range of analgesic protocols was employed, with some studies using unimodal opioid-based analgesia, others employing multimodal analgesia, and some using unspecified multimodal regimens [297,363,364,394,397,398,402,403,412,495]. Routes of administration also varied, with most studies investigating the oral route (PO). The heterogeneity of patient populations included in the studies, ranging from healthy individuals to those with various etiologies of diseases [86,495], further complicated the interpretation of the results.

Another factor contributing to the limited evidence for cannabinoids in POP relief might be the way surveys are worded. The phrasing of questions posed to patients could unintentionally influence their responses, hindering researchers’ ability to obtain accurate data relevant to their specific research goals. This is particularly noteworthy considering the findings of Khelemsky et al. [405]. Despite systematic reviews highlighting a lack of robust clinical evidence for cannabinoids in acute pain management, their study showed that patients generally perceive marijuana as at least somewhat effective for pain control. Additionally, patients expressed a willingness to use cannabinoid medications if prescribed by a healthcare provider. This discrepancy highlights the potential influence of question formulation on research outcomes.

Moreover, the selectivity of cannabinoid analogs at CB1 and CB2 receptors is crucial, which may be one of the leading causes of the therapeutic failure found in some studies, considering the opposite effects that may occur between CB1r and CB2r activation in some experimental contexts. These receptors have distinct locations and functions [97,184,496,497]. CB1 receptors are primarily located in the central nervous system, and their activation can produce psychotropic side effects [498,499,500]. CB2 receptors are abundant in peripheral tissues [25,501,502]. Their activation is associated with anti-inflammatory effects and does not show psychotropic actions [25,115,503]. These effects emphasize the importance of developing cannabinoid medications with targeted selectivity for CB2 receptors. By focusing on CB2 activation, researchers might achieve better pain relief with fewer side effects than medications that activate CB1 and CB2 receptors [18,22,227].

Finally, Pernia-Andrade et al. [504] provide compelling evidence supporting a pain-exacerbating mechanism of cannabinoid signaling in animals exposed to intense noxious stimuli. Their findings suggest that cannabinoid drugs and endocannabinoids (eCBs) produced in the spinal cord can disrupt the inhibitory regulation of pain-perceiving neurons, thereby facilitating the transmission of painful and non-painful mechanical stimuli along pain pathways to higher brain centers, effectively opening a “pain gate”.

## 8. Future Perspectives and Conclusions

The exploration of cannabinoid neuromodulation systems and the synthesis of CB1r and CB2r agonists present promising avenues for therapeutic use in pain management. Despite the demonstrated antinociceptive activity of cannabinoid agonists, particularly in preclinical studies, their clinical efficacy in the treatment of POP remains inconclusive. While some evidence suggests potential benefits, such as the avoidance of known adverse effects associated with traditional analgesics like opioids and nonsteroidal anti-inflammatory drugs (NSAIDs), the overall clinical data on cannabinoid effectiveness in perioperative and acute pain settings are limited and heterogeneous.

The current literature underscores the need for further well-designed clinical trials to elucidate the specific pathologies and conditions wherein cannabinoid agents might offer advantages over existing therapeutic options. Notably, such trials should adhere to rigorous methodological standards, as outlined by initiatives like the Initiative on Methods, Measurement, and Pain Assessment in Clinical Trials (IMMPACT), to ensure the validity and reliability of their findings [505]. Additionally, while new routes of cannabinoid administration, such as oral cannabis oil formulations, offer potential benefits in terms of safety and patient acceptability, their efficacy in the context of postoperative pain management requires robust investigation.

Furthermore, the potential long-term implications of cannabinoid therapy in reducing the incidence of chronic postsurgical pain and mitigating the need for prolonged opioid use remain largely unexplored. Large-scale, multicenter trials are warranted to comprehensively evaluate the role of cannabinoids in acute and postoperative pain relief, with careful consideration given to patient safety, optimal dosing regimens, and comparative effectiveness against established analgesic agents. Only through such concerted research efforts can we definitively determine the place of cannabinoids in the armamentarium of pain management strategies, thereby providing clinicians with evidence-based guidance for optimizing patient care in this challenging clinical domain.

## Figures and Tables

**Table 1:** Characteristics, clinical effects, and advantages and disadvantages of different routes of administration of cannabinoids.

		Pulmonary Route		Oral	Other Routes	
		Smoking	Vaporization		Oral Transmucosal	Topical
**Characteristics**		It is the most common route of administration but not recommended (joints, bongs, pipes, etc.). Combustion at 600–900 °C produces toxic byproducts: tar, PAHs (polycyclic aromatic hydrocarbons), carbon monoxide (CO), and ammonia (NH_3_). Chronic use is associated with respiratory symptoms (bronchitis, cough, phlegm) but not with lung cancer or COPD (if cannabis only). Patients may mix with tobacco, increasing respiratory/cancer risk. 30–50% of cannabis is lost in ‘side-stream’ smoke.	Heat cannabis at 160–230 °C reduces CO, but PAHs are not eliminated. Vaporization produces significantly fewer harmful byproducts than smoking. Reduced pulmonary symptoms were reported compared to smoking.	Oils, capsules and other oral routes are becoming increasingly popular due to the convenience and accuracy of dosing. Edibles (brownies/cookies) can be more difficult to dose. Juicing and cannabis teas do not allow for adequate decarboxylation of the raw plant. Tinctures and lozenges with limited research. Intermediate onset	Nabiximols oral spray is currently the only cannabis-based prescription that delivers a standardized dosage of CBD/THC in a 1:1 ratio with extensive research.	Topical is ideal for localized symptoms (dermatological conditions, arthritis), with limited research evidence.
**Clinical effects**	Onset (min)	5–10 min		60–180 min	15–45 min	Variable
	Duration (h)	2–4 h		6–8 h	6–8 h	Variable
**Advantages**		Rapid action benefits acute or episodic symptoms (nausea/pain).		Less odor, convenient and discreet, prolonged effect. Advantage for chronic disease/symptoms.	Pharmaceutical form (nabiximols) available, with documented efficacy and safety.	Less systemic effect, suitable for localized symptoms.
**Disadvantages**		Dexterity is required, vaporizers may be expensive, and not all are portable.		Titration challenges due to delayed onset.	Expensive, spotty availability.	Only local effects.

**Table 2:** Adverse events mainly associated with THC content in cannabis-based medicines.

Side Effect	Most Common	Common	Rare
**Drowsiness/fatigue**	**×**		
**Dizziness**	**×**		
**Cough, phlegm, bronchitis (smoking only)**	**×**		
**Anxiety**	**×**		
**Nausea**	**×**		
**Cognitive effects**	**×**		
**Euphoria**		**×**	
**Blurred vision**		**×**	
**Headache**		**×**	
**Orthostatic hypotension**			**×**
**Toxic psychosis/paranoia**			**×**
**Depression**			**×**
**Ataxia/dyscoordination**			**×**
**Tachycardia (after titration)**			**×**
**Cannabis hyperemesis**			**×**
**Diarrhea**			**×**

**Table 3:** Summary of clinical trials evaluating cannabinoids in postoperative pain. AZD1940, peripherally restricted CB1r/CB12r agonist; Cannador, mixture of cannabinoid plant extracts containing predominantly THC and CBD (ratio 1:0.3–0.5); CBD, cannabidiol; GW842166, selective CB2r agonist; I.M.IMtramuscular; P.O.POr os; PCA, patient-controlled analgesia; RCT: randomized controlled trial; THC, tetrahydrocannabinol.

Type and Design of the Study	Subjects	Surgical Procedure	Primary Outcome	Postoperative Analgesia	Cannabinoid Intervention	Main Results	Reference
RCT (double blind, placebo-controlled, crossover design)	56 patients	Acute Fracture or trauma	N/S	N/S	Levonantradol I.M. Preoperative regimen. Single injection: 1, 1.5, 2, 2.5, and 3 mg.	Pain relief with the four doses; analgesia persisted for more than 6 h with the 2.5 and 3 mg doses.	Jain et al., 1981 [349]
Placebo-controlled, single-dose	100 patients	Renal surgery with lumbar incision	N/S	Noramidopyrine (metamizol), Camylofine (anti-cholinergic drug)	Levonantradol I.M. Postoperative regimen. Single injection: 1 and 2 mg.	No significant difference compared with placebo.	Guillaud et al., 1983 [348]
RCT (double blind, placebo-controlled, single dose, parallel)	40 patients	Elective abdominal hysterectomy	Pain scores	Morphine PCA	THC P.O. Postoperative regimen. Single dose: 5 mg.	No significant difference compared with placebo. Increased awareness of surroundings is more frequently reported with THC	Buggy et al., 2003 [259]
RCT (double-blind, placebo-controlled)	41 patients	Orthopedic, gynecology, urology, and plastic or general surgery	Opioid consumption at 24 h	Morphine PCA	Nabilone P.O. Preoperative and postoperative regimen. 1 and 2 mg.	No significant difference compared with placebo.	Beaulieu et al., 2006 [350]
RCT (double-blind, placebo-controlled)	100 patients	Radical retropubic prostatectomy	Opioid consumption at 48 h	Piritramide PCA	Dronabinol P.O. Preoperative (evening before operation) and postoperative until the morning of the 2nd postoperative day) regimen. 8 doses of 5 mg.	No significant difference between dronabinol and placebo groups in the self-administration of post-operative piritramide.	Seeling et al., 2006 [322]
Dose escalating study	65 patients	Various major Surgeries (included orthopedic, gynecologic, urology, plastics, and general)	N/S	Morphine PCA	Cannador P.O. Postoperative regimen. Single dose: 5, 10, 15 and 24 mg.	Significant dose-related improvements in rescue analgesia requirements in the 10 and 15 mg groups. Study ended because of a serious vasovagal adverse event in a patient receiving 15 mg.	Holdcroft et al., 2006 [347]
CT (double-blind, placebo-controlled)	112 patients	Third molar tooth extraction	Pain scores up to 10 h postsurgery	500 mg acetaminophen, 15 mg codeine phosphate	GW842166 P.O. Preoperative regimen. Single dose: 100 and 800 mg.	In comparison to ibuprofen, single doses of GW842166 (100 and 800 mg) failed to demonstrate clinically meaningful analgesia in the setting of acute dental pain.	Ostenfeld et al., 2011 [353]
RCT (double-blind, placebo-controlled)	150 patients	Removal of impacted lower third molar tooth	Area under the curve pain scores	1000 mg acetaminophen	AZD1940 P.O. Preoperative regimen. Single dose: 800 µg.	No significant differences compared with placebo.	Kalliomaki et al., 2013 [352]
RCT (double-blind, placebo-controlled)	99 patients	Arthroscopic rotator cuff repair	Pain scores Patient satisfaction with pain control Opioid consumption at days 1, 2, 7, and 14	5 mg oxycodone 325 mg acetaminophen	CBD P.O. Postoperative regimen. Repeated dose (three times a day, 14 days): 25 and 50 mg	Days 1 and 2: Lower pain score in the CBD group. Higher patient satisfaction with pain control. No statistical difference between groups in opioid consumption Days 7 and 14: No significant differences compared with placebo.	Alaia et al., 2022 [422]
RCT (double-blind, placebo-controlled)	80 patients	Total knee arthroplasty	Pain and sleep scores Cumulative postoperative opioid use	1000 mg acetaminophen 300 mg gabapentin 15 mg meloxicam 5 mg oxycodone	CBD topical. Postoperative regimen. Repeated dose (three times a day, 14 days): 120 mg/ounce	No significant differences compared with placebo.	Haffar et al., 2022 [421]
RCT (double-blind, placebo-controlled)	94 patients	Ureteroscopy with stent placement for urinary stone disease	Pain scores Postoperative opioid use	5 mg oxycodone	CBD P.O. Postoperative regimen. Repeated dose (3 days): 20 mg	No significant differences compared with placebo.	Narang et al., 2023 [424]
RCT (double-blind, placebo-controlled)	83 patients (follow-up)	Arthroscopic rotator cuff repair	Pain scores Patient satisfaction with pain control Opioid consumption at 7 and 14 days	5 mg oxycodone 325 mg acetaminophen	CBD P.O. Postoperative regimen. Repeated dose (three times a day, 14 days): 25 and 50 mg	No significant differences compared with placebo.	Alaia et al., 2024 [423]

**Table 4:** Factors related to the lack of evidence regarding analgesia for postsurgical pain.

**Related to the symptoms**	Postoperative pain is usually localized. In this context, the activation of the endocannabinoid system is minor compared to that in chronic pain, and cannabinoids are mainly associated with the relief of neuropathic pain.
	Pain assessment is highly subjective, and the quantification and comparison between study groups are generally inconclusive.
**Related to the product**	On the one hand, the composition of phytocannabinoids is very heterogeneous. On the other hand, studies of its components separately do not provide the same results as when the whole plant is analyzed.
	Human clinical trials on postoperative pain used almost exclusively THC or an analog.
	Few studies are testing a mixture of THC/CBD or CBD without THC in humans for the treatment of postoperative pain.
	Lack of knowledge about cannabinoid interaction (synergic, antagonistic, entourage effect).
**Related to the administration route**	In the systemic routes, the clinical doses seem limited by the appearance of side effects, primarily psychotropic.
	There is no record of perimedullary administration at the clinical level of cannabinoid compounds, with robust and proven analgesic preclinical evidence for this route of administration.
**Related to the studies**	Short-term duration (mostly single-dose administration and a short follow-up period).
	There is considerable variation in the doses and therapeutic guidelines employed.
	There are no 100% effective standard treatments against which to compare the effect of cannabinoids.
	A small number of patients in multiple operative settings.
	No homogenous groups (healthy patients, patients with diseases of different etiologies).
